# Unraveling the Early-Life Impacts of Environmental Exposures: From Fetal Growth to Child Development

**DOI:** 10.3390/toxics13100849

**Published:** 2025-10-08

**Authors:** Wei Xia

**Affiliations:** 1 School of Public Health, Tongji Medical College, Huazhong University of Science and Technology, No. 13 Hangkong Road, Wuhan 430030, China; xiawei@hust.edu.cn; 2 Key Laboratory of Environment and Health, Ministry of Education, No. 13 Hangkong Road, Wuhan 430030, China; 3 Key Laboratory of Environmental Pollution and Health Effects, Ministry of Ecology and Environment, No. 13 Hangkong Road, Wuhan 430030, China

## 1. Introduction

The early-life period, encompassing fetal development, infancy, and early childhood, represents a window of extraordinary vulnerability to environmental influences. During this time, rapid cellular proliferation, organ formation, and the intricate programming of biological systems are highly susceptible to disruption from exogenous factors. Environmental exposures in early life—to pollutants such as heavy metals, airborne particles, organic chemicals, and pesticides—can have profound and lasting effects on fetal growth, birth outcomes, and children’s neurodevelopmental, behavioral, and emotional health. The mechanisms underlying these effects are complex and multifaceted, involving disruptions to placental function, the induction of oxidative stress and inflammation, dysregulation of the endocrine system, and alterations in epigenetic landscapes and gene expression.

This Special Issue, entitled “The Effects of Environmental Exposures on Fetal Growth, Children Development and the Corresponding Mechanisms”, aims to explore these critical issues by presenting methodologically diverse research on the impact of various environmental contaminants. The collated articles employ a range of approaches—from epidemiological cohorts and biomarker analyses to experimental models—to shed light on the pathways linking exposure to health outcomes. The evidence presented in this Special Issue provides valuable insights and may inform future public health strategies and prevention policies.

## 2. An Overview of Published Articles

The six articles published in this Special Issue collectively advance our understanding of the multifaceted impacts of environmental exposures on early life health, which can be categorized into three main research directions: identifying critical windows and vulnerable populations in epidemiological studies, elucidating underlying molecular mechanisms, and revealing potential long-term and multigenerational consequences.

Firstly, several studies underscore the importance of precise exposure assessment in human epidemiology, highlighting critical susceptibility windows, vulnerable subpopulations, and the interactions of mixed exposures. Li et al. (Contribution 1) demonstrate that maternal exposure to ozone just one month before conception and in early pregnancy significantly increases the risk of birth defects, pinpointing a previously underappreciated window of susceptibility. Their work further identifies socioeconomic factors (lower education, income, and rural residence) as key effect modifiers, emphasizing health disparities. Similarly, Huang et al. (Contribution 2) reveal that the timing of exposure is crucial for neurodevelopmental outcomes, finding adverse effects from both prenatal and postnatal phthalate exposure on behavioral problems in toddlers. Furthermore, Rosolen et al. (Contribution 3) illustrate the complexity of mixed exposures, showing that the co-exposure to high levels of lead and manganese synergistically impairs cognitive development in 18-month-old boys. Together, these studies provide a powerful framework for future public health research, stressing the need to consider the specific timing of exposure, social determinants of health, and the interactive effects of chemical mixtures.

Secondly, two papers delve into the molecular biomarkers that may mediate the link between environmental exposures and adverse health outcomes, focusing on telomere length and epigenetic regulation. Jiang et al. (Contribution 4) provide a plausible mechanism for long-term health risks by linking prenatal organochlorine pesticide exposure to shortened telomere length in cord blood—a marker of cellular aging and genomic instability. This effect was modified by infant sex and maternal BMI, again pointing to individual susceptibility. At the epigenetic level, Tsai et al. (Contribution 5) show that prenatal exposure to perfluoroalkyl substances (PFAS) can alter global histone methylation patterns (H3K4me3 and H3K27me3) in young children. This epigenetic dysregulation offers a key mechanistic insight into how early-life chemical exposures can potentially reprogram gene expression and influence disease susceptibility years later.

Finally, moving from association to causation and from a single generation to multiple, experimental research provides evidence of long-lasting harm. Przepiórska-Drońska et al. (Contribution 6) used a mouse model to demonstrate that prenatal exposure to the UV filter Benzophenone-3 (BP-3) induces neurotoxic and pro-apoptotic effects not only in directly exposed offspring (F1) but also in the subsequent, unexposed generations (F2). This multigenerational study, showing sex- and brain-region-specific effects via the Fas-mediated apoptotic pathway, delivers a warning: some environmental chemicals can disrupt brain development and compromise neurological health across generations, solidifying the imperative need to protect early-life environments.

## 3. Conclusions

Collectively, the studies in this Special Issue provide novel evidence that early-life exposure to ubiquitous environmental pollutants—including air pollutants, metals, pesticides, PFAS, phthalates, and BP-3—can disrupt fetal growth and child development through diverse pathways. The findings underscore the critical importance of specific susceptibility windows, the heightened vulnerability of certain subpopulations, and the critical role of underlying mechanisms such as oxidative stress, epigenetic reprogramming, and apoptosis. These insights move the field beyond mere association towards a more mechanistic understanding of how environmental exposures program health and disease across the lifespan.

Looking ahead, future research should continue to integrate epidemiological findings with mechanistic studies. Key priorities include the use of longitudinal cohorts to assess long-term outcomes, the application of multi-omics approaches to elucidate novel pathways, and the development of interventions targeting identified susceptibility windows and mechanisms. Such efforts are crucial to inform evidence-based public health policies and preventive strategies for protecting mothers and children from environmental threats.

## Data Availability

Not applicable.

